# Total eyebrow reconstruction with a free superficial temporal artery flap: A case report

**DOI:** 10.1016/j.jpra.2024.11.012

**Published:** 2024-11-28

**Authors:** Nguyen Van Phung

**Affiliations:** Department of Plastic and Aesthetic Surgery, Tra Vinh University, Tra Vinh, Vietnam

**Keywords:** Eyebrow reconstruction, Superficial temporal artery, Free flap, Giant congenital melanocytic naevus

## Abstract

The eyebrow plays a crucial role in facial aesthetics and expression. Reconstructing an eyebrow defect remains a challenge due to the unique characteristics of eyebrow hair. While various advanced methods exist, we report the first documented use of a free superficial temporal artery flap for total eyebrow reconstruction. This case study describes a successful eyebrow reconstruction in a patient with a total eyebrow defect. The free superficial temporal artery flap provided excellent vascularity, texture, and hair growth, mimicking the natural eyebrow. Our findings suggest that the free superficial temporal artery flap offers a promising new option for reconstructing large eyebrow defects. This technique warrants further investigation in larger patient populations.

## Introduction

The eyebrows are critical facial structures serving a multitude of functions. They play a significant role in facial recognition, emotional expression (sadness, surprise, anger), gender identification, and aesthetics. Additionally, they serve a protective role, shielding the eyes from sweat and minor injuries.^1^ Eyebrow abnormalities can significantly impact a patient's confidence and quality of life.^2^ Reconstructing an eyebrow defect presents a unique challenge in plastic surgery due to the location and characteristics of eyebrow hair. Existing techniques for eyebrow reconstruction range from simple closure to more complex procedures, including local flaps, scalp flaps, hair grafts, and composite grafts.^3^ While pedicle superficial temporal artery flaps have been used successfully for large eyebrow defects, there is a lack of documented cases utilizing free superficial temporal artery flaps for this purpose. This report presents the first documented case of total eyebrow reconstruction using a free superficial temporal artery flap. This novel approach offers a potential solution for patients with large eyebrow defects, potentially surpassing the limitations of existing techniques.

## Case report

**Figure 1:**
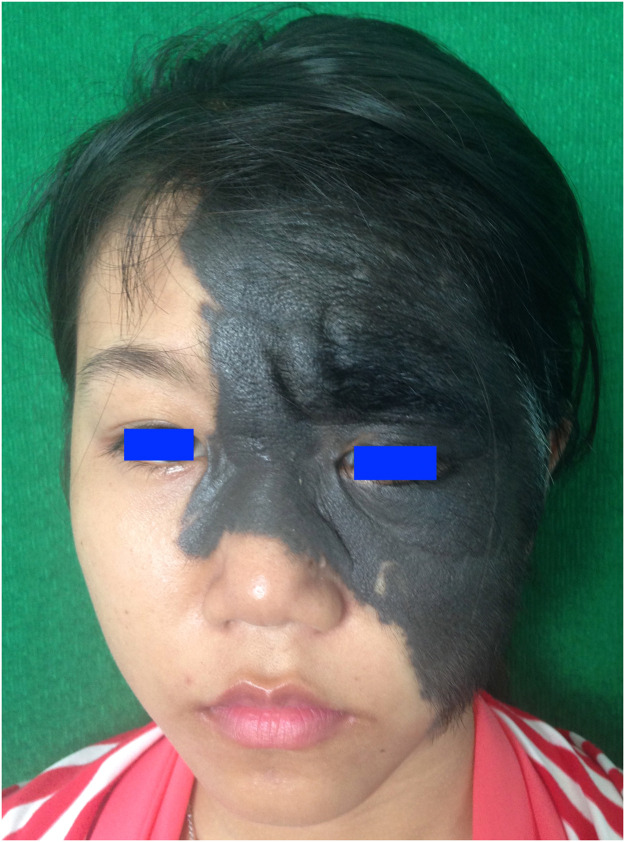
Pre-operative views: giant congenital melanocytic naevus.

**Figure 2:**
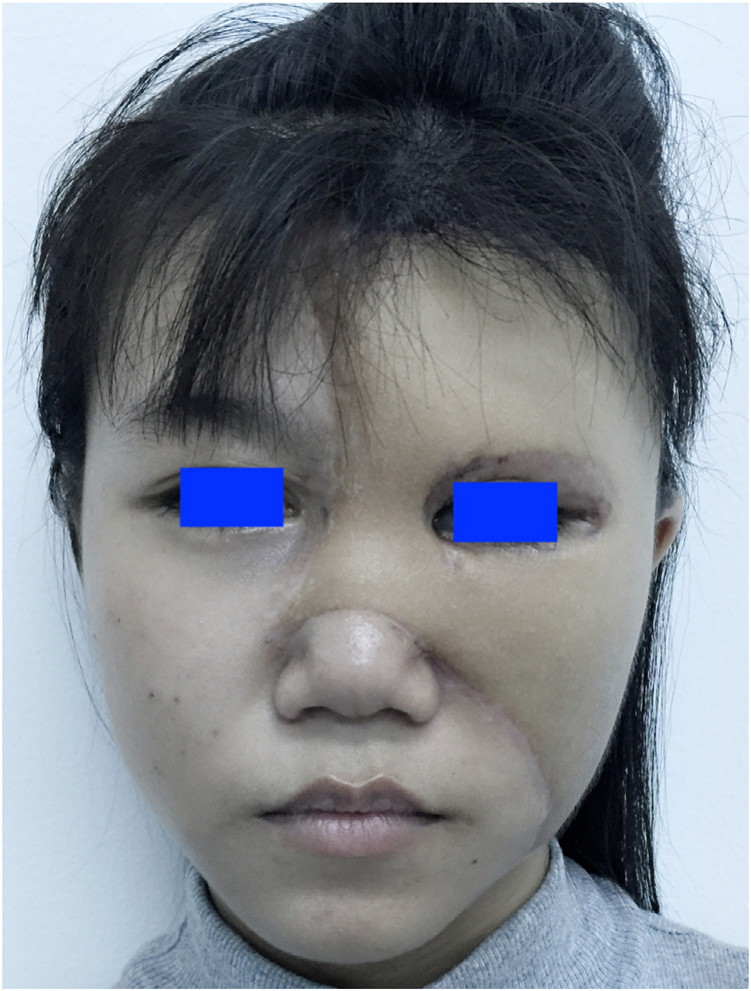
Post-tumour removal view: absence of left eyebrow.

An 18-year-old female patient presented with a giant congenital melanocytic naevus involving her left eyebrow and temporal region (Figure 1). The naevus was excised in 2015 and reconstructed with a supercharged superficial cervical artery perforator flap harvested from the scapular region. The flap's blood supply was enhanced by anastomosing the circumflex scapular artery to the superficial temporal artery (Figure 2). Six months later, she desired eyebrow reconstruction.

For eyebrow reconstruction, a free flap based on the right superficial temporal artery was chosen. Doppler ultrasound was used to preoperatively map the right and left superficial temporal arteries for the flap pedicle and recipient vessels. The flap design considered the size and hair direction of the contralateral eyebrow.

**Figure 3:**
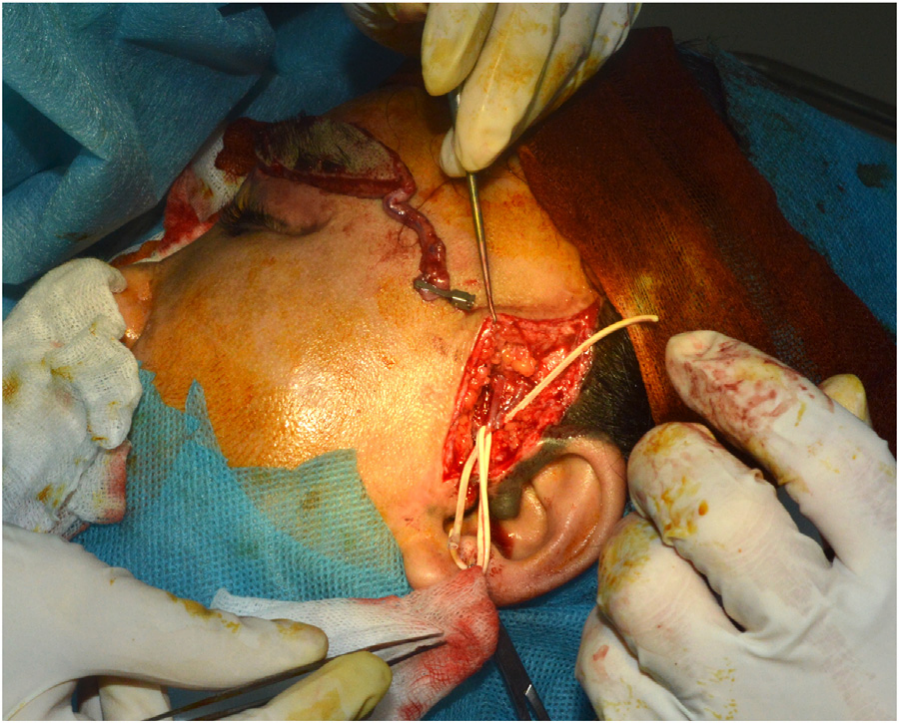
Intraoperative views.

The surgery was performed under local anaesthesia with 3.5 times magnification. Lidocaine 2 % was injected subcutaneously and intradermally to both the donor and recipient sites prior to incision and was repeated every 45–60 min as needed for patient comfort. No general anaesthesia was required. The patient was placed in a supine position. The recipient site was prepared by exposing the left superficial temporal vessels. The planned anastomosis site and the required vascular pedicle length were determined. A skin and subcutaneous tissue flap was harvested from the right temporal region based on the frontal branch of the superficial temporal artery. The dissection aimed to preserve the underlying hair follicles. The lateral branches of the artery were ligated and divided. The vascular pedicle was approximately 10 cm in length. The flap was detached and transferred to the recipient site. The superficial temporal vessels of the flap were anastomosed in an end-to-end fashion to the left superficial temporal vessels using 9–0 Prolene sutures. The flap margins were meticulously sutured to the recipient eyebrow defect using 6–0 Prolene sutures (Figure 3). Haemostasis was confirmed, and the donor and recipient sites were closed in layers. A drainage tube was placed at the reconstruction site. The total surgical time was 2 h, and a total of 400 mg of lidocaine (20 ml) was used.

**Figure 4:**
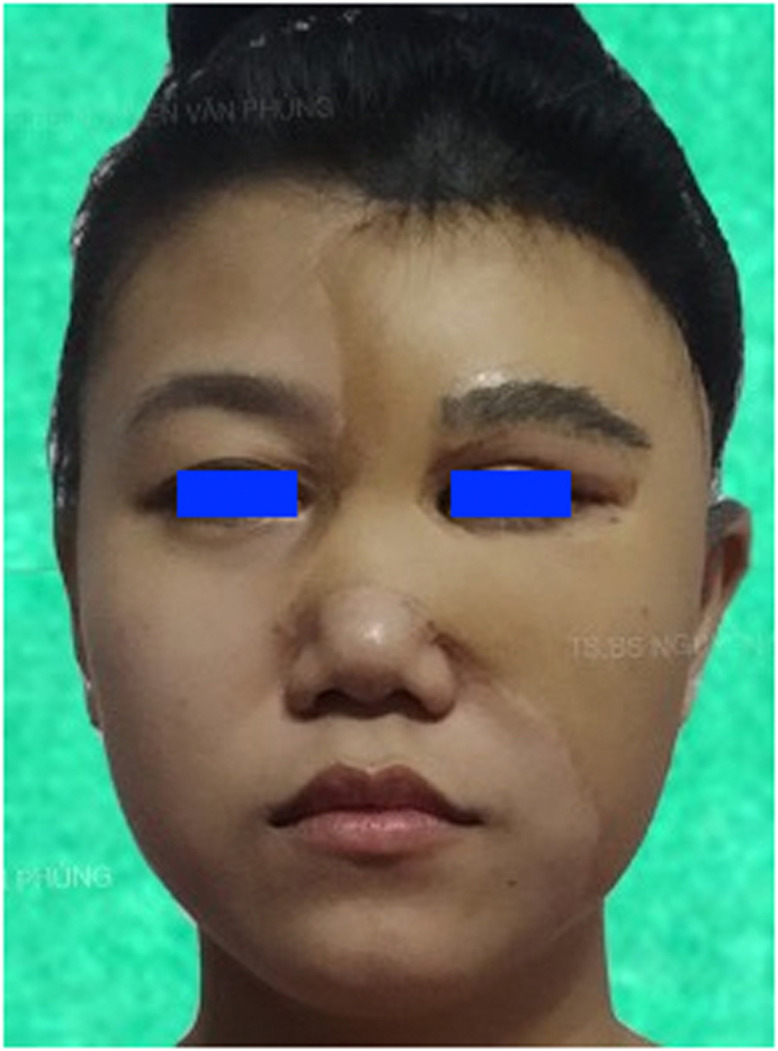
Postoperative views.

The post-operative course was without any complications. The drainage tube was removed 16 h post-operatively, and the patient was discharged home. Complete flap survival with hair growth was observed. The reconstructed eyebrow showed good stability and the patient reported high satisfaction. The reconstructed eyebrow remained stable with hair growth at over five years post-operatively (Figure 4).

## Discussion

This case report describes the successful reconstruction of a total eyebrow defect in an 18-year-old female patient following the removal of a giant congenital melanocytic naevus. While naevi are typically managed with excision, significant post-operative defects can negatively impact a patient's appearance and quality of life, particularly when facial structures like the eyebrow are involved.

Traditionally, eyebrow reconstruction employs a variety of techniques depending on the size and location of the defect. These techniques range from simple closure to more complex procedures like local flaps, scalp grafts, and follicular unit transplantation.^3^ Selecting the most suitable approach involves careful consideration of factors like the defect size and colour, eyebrow orientation, and the patient's natural features. Balancing functional and aesthetic outcomes while minimizing donor site morbidity is also crucial.^3^

For partial eyebrow defects, local flaps harvested from the uninjured contralateral eyebrow remain the gold standard due to their colour and hair growth characteristics matching the recipient site.^4^ However, recent decades have seen the rise of superficial temporal artery island flaps for eyebrow reconstruction. These flaps offer advantages like hair-bearing skin with appropriate texture and thickness.^5^^,^^6^ Other authors recommended using the advancement V-Y flap with an orbicularis oculi-based pedicle for defects less than half of the eyebrow length and the superficial temporal artery island flap for defects exceeding half of the eyebrow length.^7^

While effective, island flaps become unsuitable when the ipsilateral temporal region or blood vessels are compromised.^8^ Free flaps offer a solution in such cases. While they can be technically demanding and require longer operative times compared to island flaps, they provide unmatched versatility and can be used even when the donor site has been damaged.^9^ Notably, free superficial temporal artery flaps share the same structural and aesthetic properties as the recipient eyebrow, including hair growth characteristics. Additionally, the rich vascular supply of the superficial temporal artery allows for successful microsurgical anastomosis, enabling reconstruction under local anaesthesia with shorter hospitalization and recovery times compared to traditional free flaps.^9^ While local anaesthesia is typically sufficient, the type of anaesthesia should be discussed with patients preoperatively. The amount of lidocaine administered should be carefully calculated to prevent potential toxicities. For patients with significant surgical anxiety, sedation or general anaesthesia may be considered.

This case report presents the first documented instance of total eyebrow reconstruction using a free superficial temporal artery flap. The successful outcome, with complete flap survival and hair growth, highlights the potential of this technique as a viable option for reconstructing large or complex eyebrow defects, especially when traditional methods are not feasible. Further studies with larger patient populations are warranted to validate the long-term efficacy and refine the surgical approach.

## Funding

None.

## Patient consent

The patient provided written consent for the use of her images in the paper.

## Ethical approval

Not required.

## Declaration of competing interest

None declared.
